# Microbial interactions and the homeostasis of the gut microbiome: the role of *Bifidobacterium*

**DOI:** 10.20517/mrr.2023.10

**Published:** 2023-05-10

**Authors:** Alberto J.M. Martin, Kineret Serebrinsky-Duek, Erick Riquelme, Pedro A. Saa, Daniel Garrido

**Affiliations:** ^1^Laboratorio de Redes Biológicas, Centro Científico y Tecnológico de Excelencia Ciencia & Vida, Fundación Ciencia & Vida, Facultad de Ingeniería, Arquitectura y Diseño, Universidad San Sebastián, Santiago 8580702, Chile.; ^2^Department of Chemical and Bioprocess Engineering, Pontificia Universidad Católica de Chile, Santiago 833115, Chile.; ^3^Department of Respiratory Diseases, School of Medicine, Pontificia Universidad Católica de Chile, Santiago 7820436, Chile.; ^4^Institute for Mathematical and Computational Engineering, Pontificia Universidad Católica de Chile, Santiago 7820436, Chile.

**Keywords:** *Bifidobacterium*, colonization resistance, gut dysbiosis, microbial interactions

## Abstract

The human gut is home to trillions of microorganisms that influence several aspects of our health. This dense microbial community targets almost all dietary polysaccharides and releases multiple metabolites, some of which have physiological effects on the host. A healthy equilibrium between members of the gut microbiota, its microbial diversity, and their metabolites is required for intestinal health, promoting regulatory or anti-inflammatory immune responses. In contrast, the loss of this equilibrium due to antibiotics, low fiber intake, or other conditions results in alterations in gut microbiota composition, a term known as gut dysbiosis. This dysbiosis can be characterized by a reduction in health-associated microorganisms, such as butyrate-producing bacteria, enrichment of a small number of opportunistic pathogens, or a reduction in microbial diversity. *Bifidobacterium* species are key species in the gut microbiome, serving as primary degraders and contributing to a balanced gut environment in various ways. Colonization resistance is a fundamental property of gut microbiota for the prevention and control of infections. This community competes strongly with foreign microorganisms, such as gastrointestinal pathogens, antibiotic-resistant bacteria, or even probiotics. Resistance to colonization is based on microbial interactions such as metabolic cross-feeding, competition for nutrients, or antimicrobial-based inhibition. These interactions are mediated by metabolites and metabolic pathways, representing the inner workings of the gut microbiota, and play a protective role through colonization resistance. This review presents a rationale for how microbial interactions provide resistance to colonization and gut dysbiosis, highlighting the protective role of *Bifidobacterium* species.

## INTRODUCTION

The human gut is colonized by a dense community composed of trillions of microorganisms called the gut microbiota^[1,2]^. Such a high number of microbes influences several aspects of host health^[3]^. This community is dominated by up to 90% of two phyla: Bacteroidota and Bacillota^[4,5]^ (formerly Bacteroidetes and Firmicutes)^[6]^. Other phyla, such as Verrucomicrobiota (*Akkermansia* spp.), Actinomycetota (*Bifidobacterium* spp.), and Pseudomonadota (*Escherichia* spp.) make a smaller contribution, albeit play significant roles in this community^[7,8]^. Importantly, each phylum represents dozens of different species and strains^[9,10]^. Most of these microorganisms are commensals, but a small number of opportunistic bacteria can cause damage to the host via toxins or pro-inflammatory molecules in some specific situations and diseases^[11,12]^. In addition, other alterations in the microbiome can be associated with various types of disorders due to physiological interactions between the microbial community and human host^[12-18]^.

Each subject harbors a unique gut microbiota profile that is usually more conserved at the functional than taxonomical level^[19]^. The gut microbiota of any person may be composed of more than 500 different microorganisms^[20]^, making it one of the most complex known microbial communities. The gut microbiota shows distinct colonization patterns in newborns^[21]^, usually dominated by *Bifidobacterium* species in the first year of life, shaped by the birth and feeding type^[22]^. *Bifidobacterium* is a genus of strict anaerobes, gram-positive, and fermentative microorganisms, which are usually regarded as safe and beneficial for health. Later in life, a plant-based diet switches the microbiota to a more complex community characterized by both higher species and functional diversity^[23]^, where *Bifidobacterium* retains a significant relative abundance in the adult human gut as well as its role in health. However, its abundance decreases compared to the infant microbiota^[24]^.

Major advances have been made to understand the importance of the gut microbiota in human health. Most studies rely on 16S rRNA sequencing to provide the relative abundance profiles of this community, which are helpful in estimating microbial diversity^[1]^. However, these studies only provide a snapshot of the community and do not consider the interactions between its constituent members^[25]^. Why some microbes are more abundant than others and coexist with or exclude others are questions without obvious answers. Approximately 30,000 interactions between microbes are estimated to occur at a given time^[26]^. More complexity is added if we consider that microbes display biogeographical preferences in the gut and are present at different abundances and activity levels in different locations^[27,28]^. Complex microbial interactions dictate the composition of the microbiota in great part, but this remains poorly understood^[29]^.

*Bifidobacterium* plays a pivotal role in the gut microbiota and contributes to health through multiple activities and interactions with other gut microbes. This review aims to provide a rationale for how microbial activities and microbial interactions, especially those of *Bifidobacterium*, contribute to colonization resistance and a balanced gut microbiome composition.

### Metabolic activities of gut microbiota and Bifidobacterium

The gut microbiome is known for its dependence on the diet, where dietary fibers are major drivers in the composition of this community^[30]^. Some microbial groups in the gut are equipped with a wide enzymatic repertoire targeting almost all complex dietary polysaccharides such as pectins, xylans, fructans, starch, and arabinogalactans^[31,32]^. *Bifidobacterium* and *Bacteroides* species are the primary degraders of these polysaccharides^[25]^, and molecular mechanisms have been resolved in part. Although utilization of plant-derived oligosaccharides is common among gut microbes, recent studies have increased our understanding of the molecular adaptations of these genera to use more complex polysaccharides, especially host-derived glycans^[33]^. These findings highlight the ability of *Bifidobacterium* and *Bacteroides* to adapt to the intestinal environment. One of these complex substrates is human milk oligosaccharides (HMOs), an important carbon source for *Bifidobacterium* provided to infants via breast milk. HMOs are composed of lactose with repetitions of *N*-acetylglucosamine, fucose, and sialic acid. HMOs have a strong bifidogenic effect, which can be explained by multiple molecular adaptations in their genomes, including ABC transporters and specialized glycosyl hydrolases. The gut microbiota can also target other host-derived dietary substrates such as mucins and milk glycoproteins^[33]^. *N*- and *O*-Glycans found in IgA and mucins can be accessed and used as carbon and energy sources for bacteria such as *Bifidobacterium bifidum*, *Bacteroides thetaiotaomicron,* and *Akkermansia muciniphila*^[34]^.

Microbiome-derived metabolites influence several physiological processes within the host. The gut microbiome produces millimolar concentrations of short-chain fatty acids (SCFAs)^[35]^, such as acetate, propionate, and butyrate. Their concentrations vary in different segments of the intestine and are released in a ratio of 3:1:1 for acetate, propionate, and butyrate^[35,36]^. Other acids, such as lactate and succinate, are considered intermediates in gut microbiota metabolism and participate in cross-feeding reactions, generally absent in fecal samples^[37-39]^. *Bifidobacterium* central metabolism, the bifid shunt, theoretically produces acetate and lactate in a 3:2 ratio, together with 2.5 moles of ATP per mole of glucose^[40]^. This ratio could indeed show variations according to the dietary source. In addition, *Bifidobacterium* has been found to contribute significantly to butyrate and propionate production through different mechanisms of cross-feeding with other gut bacteria^[41-45]^. Other end-products, such as ethanol, succinate, and formate, are commonly produced by these species. For instance, the fermentation of fucose by *Bifidobacterium* results in formate production in the infant gut^[39]^. Recently, aromatic lactic acids derived from infant-associated *Bifidobacterium,* such as indole lactic acid, were found to have a strong immunomodulatory effect on CD4+ T cells by activating the aryl hydrocarbon receptor, AhR^[46]^.

SCFAs maintain host intestinal homeostasis because of their anti-inflammatory and protective effects on the intestinal epithelium, and participate in the regulation of multiple cellular processes^[4,47,48]^. Acetate is absorbed by the epithelium and reaches systemic micromolar concentrations. Propionate is primarily used in the liver^[35]^. Butyrate is the primary energy source for the colonic epithelium^[49,50]^ and its utilization by host cells requires oxygen, thereby contributing to luminal anaerobiosis^[49]^. Additionally, butyrate is an epigenetic regulator that inhibits histone deacetylases in colonocytes^[51]^ and suppresses inflammatory pathways via G-protein-coupled receptors^[52]^. Butyrate can be synthesized by four distinct metabolic pathways. Most butyrate-producing bacteria (BPB) contain butyrate kinase or butyryl-CoA: acetate-CoA transferase^[53]^. Moreover, BPB are considered critical species in the gut microbiota and essential for its stability and function^[54-56]^. BPB includes microorganisms from unrelated genera, representing a more functional than taxonomic category^[57]^. Representative BPB include *Anaerostipes caccae*, *Roseburia intestinalis*, *Lachnoclostridium symbiosum*, *Faecalibacterium prausnitzii*, *Clostridium saccharolyticum* among others^[58,59]^. BPB are highly oxygen-sensitive Gram-positive bacteria^[41]^ that, while capable of using simple oligosaccharides, appear to prefer molecules such as lactate, succinate, or acetate to produce butyrate^[54,60]^. Although BPB have beneficial effects, and a decrease in their abundance can be an indicator of declining intestinal health and response to microbial diseases^[12,61]^, the role of butyrate in host physiology has been controversial due to conflicting evidence in the literature. Variations in diet, gut microbiota composition, and individual genetic differences may also play a role in determining the effects of butyrate in a dose-dependant manner^[62-64]^. Therefore, further studies are required to determine the full scope of its effects.

### Barrier effect and gut dysbiosis

Since birth, the gut microbiome influences host responses, shaping the immune system^[65]^ and contributing to organ and tissue development, especially in the gastrointestinal tract (GI)^[66]^. The gut microbiota is one of the main contributors to the barrier effect^[67]^ that prevents the translocation of microbial cells and toxins^[55,56]^. Under normal conditions, the intestinal mucosa creates a dense barrier between the luminal compartment and the intestinal epithelium. Other effectors contribute to the barrier effect, such as immune cells and cytokines, tight junctions, secretion of antimicrobial peptides (AMPs), and mucins^[67]^.

**Figure 1:**
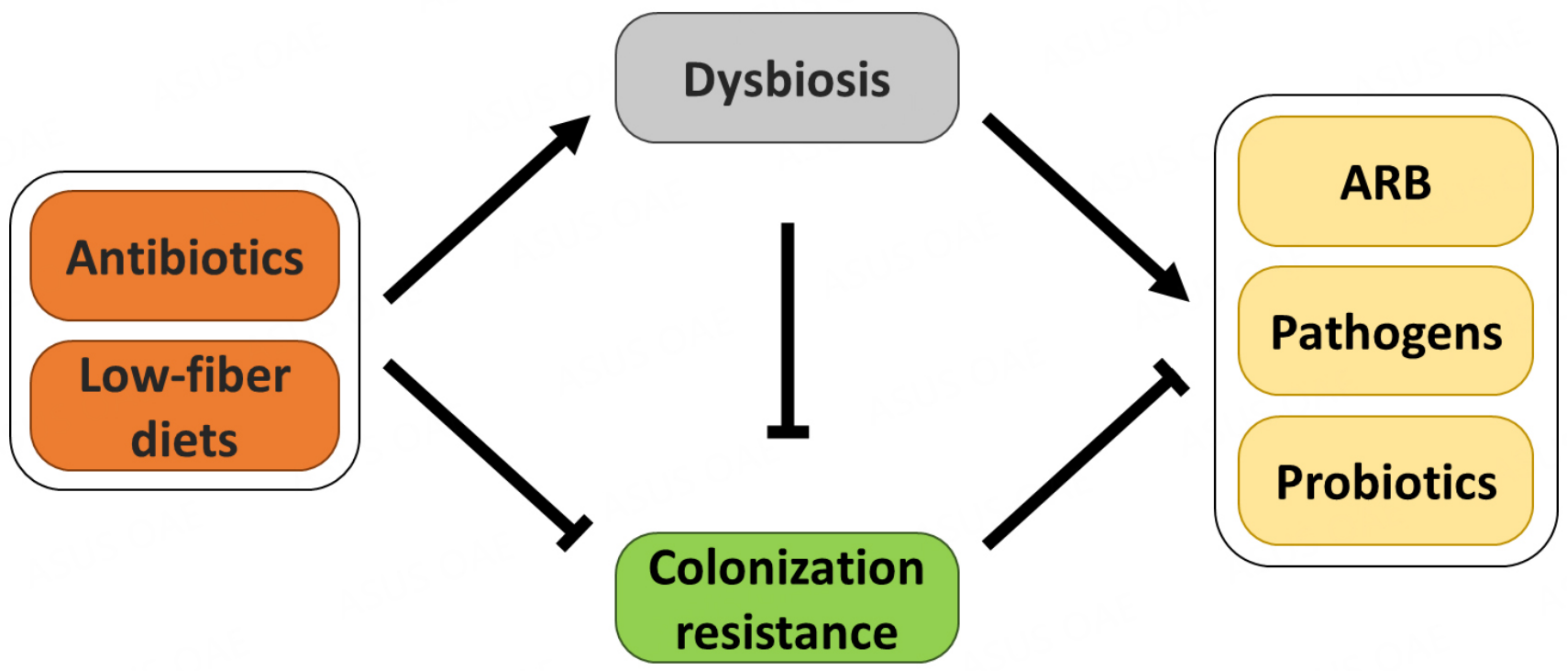
Schematic diagram of factors leading to gut dysbiosis and loss of colonization resistance. Antibiotics and diets poor in fiber have been shown to promote gut dysbiosis, reducing the ability of the epithelium to counteract pathogens and foreign bacteria, that is, colonization resistance. While a robust epithelium and gut microbiome usually inhibits the colonization and growth of potentially harmful microorganisms and probiotics, dysbiosis favors the colonization of antibiotic-resistant bacteria (ARB) and pathogens.

A healthy equilibrium between the gut microbiota species, its microbial diversity, and its metabolome is required for intestinal health, promoting regulatory or anti-inflammatory immune responses^[52,65]^. In contrast, the loss of this equilibrium due to antibiotics or a low-fiber diet results in alterations in the gut microbiota composition, a term known as gut dysbiosis [Figure 1]^[68]^. This microbial condition is characterized by different microbial changes^[12]^, and several studies have highlighted the contribution of gut dysbiosis to many chronic diseases, including type 2 diabetes, inflammatory bowel diseases (IBD), and cardiovascular diseases, and other diseases like neurological conditions, cancer, among others^[12-18]^. Sometimes, gut dysbiosis is characterized by an overabundance of opportunistic pathogens, which in robust microbiota have no chance to colonize^[61,69,70]^. Some examples include toxin-producing gut microbes such as *Clostridioides difficile*, *Escherichia coli*, or *Fusobacterium nucleatum*^[71,72]^. These pathogens are generally present in very low numbers in the microbiota; however, certain external conditions favor their growth and damaging activities, contributing to colorectal cancer^[12,73]^ among other diseases. Dysbiosis also can be characterized by a depletion in health-associated microorganisms such as BPB, as is the case of IBD^[12,61,70]^. Finally, in some cases, dysbiosis is characterized by a significant rearrangement in the microbiota composition, as observed in diarrhea^[12]^. In many diseases and dysbiotic conditions, there is reduced microbial diversity, usually measured as alpha-diversity^[74,75]^; however, a reduced alpha-diversity is not always a reliable indicator of disease-associated dysbiosis. In fact, some studies have shown an inconsistent relationship between alpha diversity and non-diarrheal diseases^[12,76]^.

An imbalance in the gut microbiota, resulting in the loss of beneficial commensal microorganisms or the gain of opportunistic pathogens, is often associated with an alteration in the correct functioning of the immune system^[77]^. Gut dysbiosis favors pro-inflammatory systemic immune responses, which may lead to inflammatory diseases^[78]^. These alterations result in increased permeability, which permits the translocation of microbial products and cells, resulting in an impaired gut barrier^[74]^.

*Bifidobacterium* species play an important role in the gut microbiome by contributing to the barrier effect, maintaining the balance of the gut microbiome, and preventing pathogenic overgrowth^[79-82]^. Some species within this genus support mucosal integrity, preventing harmful substances from penetrating the body, as has been demonstrated for several bifidobacteria^[83-87]^. The barrier effect is also promoted by certain SCFAs, such as acetate and propionate, and by multiple effectors found in these species, such as pili and exopolysaccharide^[86,88]^. Finally, immune modulation by *Bifidobacterium* promotes balanced immune responses and maintains gut homeostasis^[89]^.

### Representative microbial interactions in the gut microbiome

Ecological rules dictate microbiome composition, activity, and interactions with the host^[90]^. As part of a complex host-associated ecosystem, the gut microbiome displays emergent properties that differ from those of its single constituent species. Competition for nutrients and space, microbial inhibition, and resource sharing are common interactions in the gut^[25]^. Oxygen availability, pH, peristaltic movements, and host secretions are strong environmental factors that shape microbiome composition and explain colonization preferences for the lumen, epithelium, or along the GI tract^[91]^. Gut microbes engage in multiple interactions, some of which could be positive, such as the exchange of useful metabolites, or negative, such as the competition for nutrients or the release of antimicrobials. Relevant examples are presented below.

***Cross-feeding*:** Some microbes specialize in the degradation of complex carbohydrates, such as xylans, pectins, or fructans, whereas others prefer to ferment simple carbohydrates^[92,93]^. Other microbes thrive by fermenting proteins or fatty acids, which typically release toxic molecules such as H_2_S or NH_3_^[30,73]^. Metabolic cross-feeding, which corresponds to the bacterial exchange of metabolites, is a dominant interaction in the gut microbiome that engages in a dense four-stage metabolic interaction network^[25,94,95]^. Cross-feeding can be bidirectional (both microorganisms share one or more resources) or unidirectional^[25,96]^. The degradation products of different macromolecules can be released by one bacterium and utilized by other microbes. There are several examples of cross-feeding among *Bifidobacterium* species^[41-45,97-100]^. Constituent monosaccharides are generally released as part of the consumption mechanism of these bacteria, providing them with the opportunity to cross-feed with other bacteria. For example, *Bifidobacterium bifidum* releases sialic acid and fucose during the consumption of human milk oligosaccharides and mucin, which can be consumed by *Bifidobacterium breve*, thereby facilitating its growth^[99]^. Most *B. breve* strains do not have the machinery for complex HMO utilization. However, they can be dominant and found in high numbers in the infant gut. Similarly, mucin glycans degraded by *B. bifidum* promote *Eubacterium hallii* butyrate production^[45]^.

Another type of cross-feeding occurs when SCFAs or other organic acids are exchanged. Molecules such as acetate, lactate, and succinate are end-products of the metabolism of bacteria such as *Bifidobacterium* and *Bacteroides* spp.^[101]^. These acids are commonly imported and incorporated by other species as carbon and energy sources^[31]^. Proteolysis of dietary peptides generates amino acid competition between gut microbes, resulting in the altered production of branched SCFAs^[73]^. Most BPB produce butyrate from acetate or lactate^[102]^, and certain *Clostridium* species can use lactate or succinate for butyrate production^[102]^. *Anaerostipes caccae* releases fivefold more butyrate from lactate than glucose^[54]^. *Lachnoclostridium symbiosum* uses lactate and succinate derived from *Phocaicola dorei* to increase its growth and produce butyrate^[37]^. Several *Bifidobacterium* species have been shown to promote BPB growth and butyrate production. This process is both strain- and substrate-dependent. For example, *Faecalibacterium prausnitzii*, a dominant BPB, can cross-feed with *Bifidobacterium adolescentis* and *Bifidobacterium catenulatum* when using inulin as a substrate, both *in vitro* and *in vivo*^[100]^. In addition, during HMO utilization, *B. infantis* enhanced *Anaerostipes caccae* growth via HMO degradation products, as well as acetate and lactate production^[44]^.

However, cross-feeding is not always positive. Some degradation products can be used for other commensals and opportunistic pathogens sharing similar nutritional preferences^[103]^. Another example is dietary deprivation, which is known to turn the microbiota’s metabolic activity toward utilizing host-derived glycans like mucins. Mucin glycans are rich in fucose and sialic acid, which are also used as cross-feeding metabolites^[104]^. This degradation results in microbiome-mediated erosion of the mucosal barrier and disruption of the barrier function^[104]^. This disruption permits lethal colonization of *Citrobacter rodentium* in mice, which under normal conditions does not cause a major infection^[104]^. These findings highlight the importance of diet in dysbiosis^[104]^.

***Exploitative competition*:** Some gut microbes, especially those that are taxonomically related, share similar niche preferences and therefore engage in competition^[29]^. Exploitative competition is a negative microbial interaction defined by limited resources resulting in reduced microbial growth^[105]^. Many gut microbes use simple saccharides, which are highly demanded, resulting in competition. Competition for limited nutrients results in pathogen starvation^[72,106]^. The intestinal lumen is an anaerobic environment, but oxygen diffusion near the epithelium results in microaerophilic conditions^[27]^. Pathogenic enterobacteria, such as *Shigella flexneri,* face strong competition from commensal microbes for oxygen, which is critical for their expansion^[78,107]^.

Gut commensals promote balanced immune responses and have a large arsenal of molecules that control pathogenic growth^[72]^. In contrast, pathogens such as *S. typhimurium* take advantage of a disrupted microbiota to temporally colonize the host^[108]^. Its infection causes mild intestinal inflammation that results in macrophage activation and the production of radical oxygen species (ROS) and AMPs, disturbing the stability of the microbiota and reducing the commensal population^[72]^. Some ROS, such as tetrathionate and thiosulfate, provide a competitive advantage to this pathogen by using them as alternative electron acceptors in anaerobic respiration^[109,110]^. Therefore, inflammation is a mechanism by which some pathogens disrupt colonization resistance. Salmonella-induced inflammation increases epithelial oxygenation by depleting BPB^[107]^. Antibiotic treatment also depletes commensal BPB, decreasing luminal butyrate concentrations^[111]^. The loss of BPB caused by antibiotics or dysbiosis explains the reduced butyrate absorption and increased epithelial oxygenation. Higher intestinal oxygen concentrations favor the expansion of facultative anaerobes in the gut, such as *S. typhimurium*^[111]^.

***Interference competition*:** It occurs when one or more microbes display antimicrobial activity against others. Genes participating in this process are abundant in the genomes of gut microbes^[24]^, and the gut microbiome has been described as a *warzone*^[78]^. Microcins are produced by Gram-negative bacteria, and lantibiotics or bacteriocins are characteristic of Gram-positive bacteria. Microcins are found in 34% of sequenced *Escherichia coli* strains, which might contribute to their establishment in the gut microbiota^[112]^. Some bacteriocins have practical applications in food safety^[113]^, and some have inhibitory activity against important pathogens such as *C. difficile*^[114,115]^.

Several bacteriocins have been identified in the *Bifidobacterium* spp.. They usually have low molecular weight (less than 10 kDa) and a wide range of acid and thermal stability, with Gram-positive bacteria as their primary targets^[116]^. Bifidocin A is produced by *B. animalis* and displays strong activity against *Listeria monocytogenes* by acting on its cell membrane level^[117]^. Bifidocin LHA, produced by *B. adolescentis,* inhibited *Pseudomonas aeruginosa* in a corneal infection model^[118]^. Bifidin I produced by *B. infantis* BCRC 14602 inhibits several Gram-positive bacteria, including lactic acid bacteria. A lantibiotic in *B. longum* displays strong inhibitory activity against *Clostridium perfringens* and *Bacillus subtilis*^[116]^.

In addition to their participation in cross-feeding interactions, SCFAs produced after fiber fermentation inhibit some microbes, including pathogens^[78]^. Being weak acids, SCFAs lower luminal pH and may enter bacterial cells as protonated acids, disrupting the intracellular pH. Acetate is a preserving agent, and *Bifidobacterium longum* inhibits pathogenic *E. coli* via acetate^[119]^. Gut microbes are sensitive to pH and adjust their habitats to achieve their optimum pH for growth. *Bacteroides* spp. are well known to prefer pH values of approximately 6.5, with limited growth at acidic conditions^[120]^. Butyrate reduces the expression of Type III Secretion Systems (T3SS) in *S. typhimurium* mediated by the change in pH. Butyrate also inhibits *Bacteroides* spp. in a strain- and glycan-dependent manner^[121]^. Similarly, propionate inhibits *Salmonella* growth by the same mechanism^[122,123]^.

### Colonization resistance

Colonization resistance is a fundamental property of gut microbiota for preventing and controlling infections^[74,112]^. This community poses a strong blockade against foreign microorganisms such as GI pathogens, antibiotic-resistant bacteria (ARB), and even probiotics [Figure 1]^[112]^. This property depends on a stable and healthy balanced microbiota^[72]^. It is based on direct mechanisms, including competition for nutrients, niche exclusion, or the release of toxic substances, and indirect mechanisms, such as the induction of host immune responses^[72]^. Some pathogens have developed counterstrategies to overcome colonization resistance, and the temporary loss of colonization resistance results in the expansion of certain pathogens^[72]^.

A diverse microbiota provides protection against *Listeria monocytogenes* (Lm)^[124]^. This foodborne pathogen causes severe diseases in immunocompromised individuals. Antibiotic-mediated depletion of gut commensals reduces colonization resistance and increases Lm colonization^[124]^. Animals require a high infective dose of Lm to develop an infection, which is reduced to only a few cells when treated with antibiotics^[124]^. A consortium of four microbes displayed antilisterial activity in germ-free animals, stimulating resistance to colonization against Lm^[124]^. These consortia included *Blautia producta* and *Clostridium* spp.. *B. producta* has also been implicated in other antimicrobial activities^[125,126]^. Vancomycin-resistant enterococci (VRE) is a multidrug-resistant microorganism that can colonize the human gut and cause bloodstream infections, especially after antibiotic therapy. The gut microbiota mounts resistance to colonization by VRE and limits its colonization^[125]^. Using a reductionist approach, a specific consortium of four gut microbes was found to confer VRE resistance in animals. This consortium displayed cooperative interactions; two Bacteroidales species possessed endogenous lactamase activity, allowing *Clostridium bolteae* and *Blautia producta* to clear VRE from the intestine. It was shown that to support colonization of the murine intestine by *B. producta*, the presence of the other species in the consortium and multilevel cooperation between them was necessary^[125]^*.* Later, it was found that *B. producta* produces a lantibiotic similar to nisin against VRE^[126]^. This study showed how interspecies cooperativity is important for colonization resistance^[125]^.

Excessive antibiotic use appears to be a risk factor for certain chronic diseases^[127,128]^. Antibiotics are known to cause significant perturbations in the gut microbiota^[112,129]^ and promote dysbiotic states. The extent to which an antibiotic alters the microbiota depends on the spectrum of the antibiotic, dose, and duration of administration^[112]^. Antibiotic use for extended periods opens a window of opportunity to acquire ARB through the loss of colonization resistance [Figure 1]^[74]^. Resistant bacteria are generally present in the gut microbiota but at very low levels^[74,115]^, and antimicrobial therapy increases ARB selection^[112]^. Moreover, hospitalization results in significant exposure to ARB^[112]^. Similarly, germ-free or antibiotic-treated animals develop severe infections compared to conventional animals, such as *Salmonella* enterica serovar Typhimurium or *Listeria monocytogenes,* owing to the lack of colonization resistance provided by the microbiome^[72]^.

Probiotics belonging to *Lactobacillus* and *Bifidobacterium* have a long history of use in foods and supplements, contributing to the balance of the gut microbiota^[130,131]^. There are several applications where these probiotics are recommended, such as infant colic, allergies, and antibiotic administration^[132]^. Colonization resistance limits the growth of probiotic bacteria, which usually only transit through the GI tract; permanent colonization is uncommon for probiotics^[133]^. Moreover, transient colonization is highly individualized during the consumption of probiotics^[134]^. Usually, probiotic applications do not consider colonization resistance or probiotic interactions with other members of the microbiota, which outnumber probiotics by at least 1,000 times^[74]^. Some studies have suggested that colonization is not necessary for its effects on the host^[132]^.

## CONCLUSIONS

Microbial interactions represent the inner connections of the gut microbiota and contribute to its protective role through colonization resistance against pathogens, ARB, or probiotics. Antibiotics and a low-fiber diet play a role against colonization resistance, resulting in dysbiosis with a concomitant reduction in BPB and an increased chance of colonization by foreign microbes. *Bifidobacterium* species are key members of the gut microbiota and participate in multiple cross-feeding interactions with species of the same genus and other distant species, for example, by sharing SCFAs or monosaccharides. While there are few examples showing how some bifidobacteria display beneficial effects to the host and a balanced gut ecosystem, the mechanisms, microbial interactions, or metabolites involved in their protective role are largely unknown and remain the subject of future studies.

## DECLARATIONS

### Copyright

© The Author(s) 2023.
